# Ante natal care (ANC) utilization, dietary practices and nutritional outcomes in pregnant and recently delivered women in urban slums of Delhi, India: an exploratory cross-sectional study

**DOI:** 10.1186/s12978-015-0008-9

**Published:** 2015-03-20

**Authors:** Suparna Ghosh-Jerath, Niveditha Devasenapathy, Archna Singh, Anuraj Shankar, Sanjay Zodpey

**Affiliations:** Indian Institute of Public Health-Delhi, Public Health Foundation of India, Plot No 47, Sector 44, Institutional area, Gurgaon, 122002, Haryana India; Department of Biochemistry, All India Institute of Medical Sciences, Ansari Nagar, New Delhi, 110029 India; Department of Nutrition, Harvard School of Public Health, 665 Huntington Avenue, Boston, MA 02115 USA

**Keywords:** Pregnancy, Antenatal care, Nutritional status, Nutrition counselling, Dietary intake

## Abstract

**Background:**

Antenatal Care (ANC) is one of the crucial factors in ensuring healthy outcomes in women and newborns. Nutrition education and counselling is an integral part of ANC that influences maternal and child health outcomes. A cross sectional study was conducted in Pregnant Women (PW) and mothers who had delivered in the past three months; Recently Delivered Women (RDW) in urban slums of North-east district of Delhi, India, to explore ANC utilization, dietary practices and nutritional outcomes.

**Methods:**

A household survey was conducted in three urban slums to identify PW and RDW. Socio-economic and demographic profile, various components of ANC received including nutrition counselling, dietary intake and nutritional outcomes based on anthropometric indices and anaemia status were assessed. Socio-demographic characteristics, nutrient intake and nutritional status were compared between those who availed ANC versus those who did not using logistic regression. Descriptive summary for services and counselling received; dietary and nutrient intake during ANC were presented.

**Results:**

Almost 80% (274 out of 344) women received some form of ANC but the package was inadequate. Determinants for non-utilization of ANC were poverty, literacy, migration, duration of stay in the locality and high parity. Counselling on nutrition was reported by a fourth of the population. Nutrient intake showed suboptimal consumption of protein and micronutrients like iron, calcium, vitamin A, vitamin C, thiamine, riboflavin niacin, zinc and vitamin B12 by more than half of women. A high prevalence of anaemia among PW (85%) and RDW (97.1%) was observed. There was no difference in micronutrient intake and anaemia prevalence among women who received ANC versus who did not.

**Conclusions:**

Pregnant women living in urban poor settlements have poor nutritional status. This may be improved by strengthening the nutrition counselling component of ANC which was inadequate in the ANC package received. Empowering community based health workers in providing effective nutrition counselling should be explored given the overburdened public health system.

## Background

Antenatal Care (ANC) is the key entry point for pregnant women to receive a broad range of health promotion and prevention services. WHO recommends a minimum of four ANC visits, ideally at 16, 24–28, 32 and 36 weeks and recommends health promotion including nutrition counselling as one of its important components besides others [1]. It has been shown that women attending regular ANC exhibit better knowledge, attitudes and antenatal practices compared to those not availing in several developing countries [2-4]. Nutrition education and counselling is a widely used strategy to improve the nutritional status of women during pregnancy that significantly influences foetal, infant and maternal health outcomes.

Systematic reviews on impact of antenatal dietary advice, nutrition education and counselling with or without nutrition supplementation report improved dietary intake and weight gain in mothers, reduced risk of anaemia and preterm delivery, increased head circumference and birth weight [2,5,6]. In spite of its known merits, Information, Education and Communication (IEC) during ANC along with nutrition and diet education is reported to be poorly executed and ANC is considered as a missed opportunity for IEC [7].

The availability of, and access to antenatal care is very variable across India with the key determinants being place of residence (urban/rural), socio-economic and several other cultural factors [8]. In the context of urban environment in India, there is a large inequity in access to *quality* ANC services. Economic and logistic barriers put the secondary care and private sector facilities out of reach of most poor urban residents [9], which could be the reason for most people living in urban poor settlements in India typically accessing the public health facilities for ANC [10]. Public sector urban health delivery system accessed by the underprivileged is far from adequate owing to high population to heath centre ratio, inadequately skilled staff, high staff turnover and absenteeism [11,12]. The need for strengthening the health education component of care in healthcare delivery system catering to the underprivileged that can engender changes in attitude and practice has been emphasized [13,14]. In the context of urban health system we could not locate studies in literature that quantify the counselling component provided during ANC at a health care facility. Studies have shown that pregnant women in urban poor settlements in Delhi had a poor dietary intake with majority of women consuming less than 50% of recommended dietary allowances for protein, iron and vitamin A with their intake not being significantly different from their non-pregnant counterparts [15,16].

AnteNatal and Child Health care in Urban sLums (ANCHUL) project aimed to develop, implement and evaluate the effectiveness of a complex intervention targeted at the community based health workers called Accredited Social Health Activists (ASHA) in increasing institutional delivery and improving maternal, neonatal and child healthcare practices in urban poor settlements of Delhi. During the formative phase of the project, a situational analysis was conducted and in this paper we present the findings on the availability and utilization of ANC services with a special focus on counselling, explore factors associated with ANC utilization and describe the nutrient adequacy of the diet consumed by the pregnant and recently delivered women along with their nutritional assessment.

## Methods

### Study setting

A cross sectional survey was conducted in three urban slums in India’s national capital, Delhi, between November 2010 and March 2011. The detailed description of the study area and sampling method are described elsewhere [17]. Briefly, the three urban poor clusters namely CPJ, Buland Masjid and Chanderpuri were from three constituencies of North-East district of Delhi. This district is home to the highest number of slum dwellers compared to other districts of Delhi state.

### Study population

All households (HHs) were approached to elicit information on socio-economic and demographic characteristics. During the survey we obtained a list of Pregnant Women (PW) and mothers who had given birth in the past 3 months (Recently Delivered Women (RDW)). These women were approached after 2 weeks of the initial household survey to collect detailed information about their ANC care, child birth, dietary practices and nutritional assessment. The study protocol was approved by Health Ministry Screening Committee of Government of India, institutional ethics committees of the Public Health Foundation of India, All India Institute of Medical Sciences, New Delhi, WHO Geneva and Harvard School of Public Health, Boston.

### Sample size

The sample size for the survey was calculated for measuring the prevalence of institutional deliveries in urban slums of Delhi. For this outcome to be measured with 10% relative precision assuming 33% prevalence of institutional delivery in the urban poor of Delhi [18] we needed to interview 780 women who had given birth in past one year. To identify at least 750 mothers who gave birth in past one year we needed to cover a population of 30,000 which is approximately 6000 households (assuming a crude birth rate of 25/1000).

### Data collection

The household (HH) survey was initiated only after obtaining consent from the cluster guardians. Lane to lane mapping exercise of the area ensured that all households were covered. During the mapping exercise the number of functional clinics and health posts were recorded irrespective of their registration status. From the PW and RDW who consented and were available on our revisit, information regarding utilization of ANC services and details of services offered during ANC visits were elicited by trained field interviewers using paper based forms. For assessing their dietary intake pattern, a diet survey using a 24 hour dietary recall for 2 consecutive days along with a food frequency questionnaire was conducted by trained nutritionists. Nutritional status was assessed by anthropometry (height and weight) using standard techniques and through biochemical assessment of haemoglobin status using the standard protocol for cyanmethemoglobin method. To gain an estimate of the weight gain trends in the pregnant women, a repeat weight measurement was taken after 2 weeks of the first measurement. Double data entry was done in a database designed in Microsoft Access with inbuilt validation checks. The food frequency was double entered in Microsoft Excel. The dietary recall which was taken as cooked weight from the respondent was converted into actual weights of raw foods consumed. The raw foods were then entered and converted into nutrients using a validated software DIET SOFT version 1.1 (Profound Technical Solutions, New Delhi) which utilizes values from Nutritive Value of Indian Foods [19].

### Data analysis

All data describing the socio-demographic profile and ANC utilization were summarized using descriptive statistics. The mean daily intake of the participants were computed and then compared with Recommended Dietary Allowances (RDA) for Indians [20]. In order to assess the diet quality, the adequacy of nutrient intake by each subject was computed in terms of Nutrient Adequacy Ratio (NAR) using NAR = Participant’s nutrient intake of a day/ RDA of the respective nutrient. This data was further categorized into three groups (adequate (≥1.00), fairly adequate (0.66 − <1.00) or inadequate (<0.66) NAR for various nutrients). The adequacy of consumption of various food groups were assessed using the 24 hour dietary recall data and compared with recommended intakes [21]. All women based on their pregnancy status (pregnant /lactating) were classified into anaemia grades as per the WHO classification; (For pregnant women: Haemoglobin of >11 g/dl = no anaemia, 10–11 g/dl = mild anaemia. For Lactating women > 12 g/dl = no anaemia, 10–12 g/dl = mild anaemia and 7–10 g/dl = moderate anaemia, < 7 g/dl = severe anaemia for both groups) [22]. Body Mass Index (BMI) (Weight in kg/ height in metres) was calculated for only the RDW to categorize them into (nutritional grades of underweight or Chronic Energy Deficiency (CED) with BMI <18.5 kg/m^2^, normo-weight 18.5-23 kg/m^2^ and overweight or obese >23 kg/m^2^) as per WHO [23]. The weight gain in PW was assessed by comparing them with Indian standards for recommended weight gain in trimester 2 (60 g/day) and trimester 3 (54 g/day) of pregnancy [20].

A comparison of demographic characteristics between those who availed ANC and those who did not was done using simple logistic regression. Adjusted analysis using logistic regression was performed with addition of all socio-demographic characteristics. Socioeconomic categories were derived from household income, assets and dwelling characteristics using principal component analysis. Crude and adjusted OR with 95% CI are presented along with two-sided p- values (p-value <0.05 was considered statistically significant). All data were analysed using Stata 13 [24]. To explore for possible selection bias due to high non-response rates, statistical comparison of the household demographic information between responders and non-responders was done using Pearson chi-squared test for categorical variables and student *t*-test for continuous variable.

## Results

**Figure 1:**
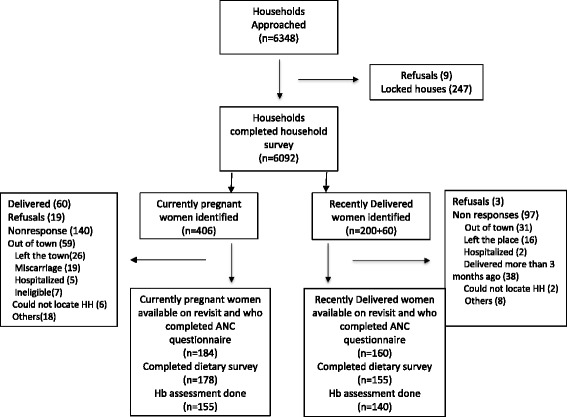
**Recruitment summary of target population.**

Of the 6348 households in the three defined areas, 6092 (96%) households participated in the HH survey. From a total population of 32,034, we identified 406 self-declared pregnant women and 200 mothers who had given birth in past three months. Of these only 184 pregnant women and 160 mothers participated in the detailed survey. The key reason for low response rate when they were approached after 2 weeks, was due to women moving to their maternal home for child birth. The study recruitment summary is elaborated in Figure 1. Socio-economic and demographic characteristics of the households of the study population is described in Table 1. There was no significant difference in household characteristics between those who were available for the detailed interview and those who were not.. The mean age was 24 years with most (86.2%) reported having married after attaining 18 years of age and 65.4% living in nuclear families (Tables 1 and 2). Half of the women were illiterate and almost all (97.7%) were homemakers.

**Table 1:** **Characteristics of the households**

**Characteristics**	**CPW and RDW who participated (n = 344)**	**CPW and RDW who did not participate (n = 261)**	**P-value**
Nuclear family (%)	225 (65.4)	181 (69.4)	0.31
Spoken language Hindi	298 (86.6)	230 (88)	0.21
Muslim (%)	254 (73.84)	200 (76.6)	0.43
Caste			
General	160 (46.5)	105 (40.2)	
OBC	139 (40.4)	117 (44.8)	0.30
SC/ST	45 (13.1)	39 (14.9)	
Family size (SD)	5.43 (2.7)	5.5 (3.1)	0.72
Durable housing (%)	274 (79.6)	213 (81.6)	0.45
Toilet facility within HH (%)	274 (79.6)	222 (85.1)	0.23
Head of household			
Male (%)	299 (86.9)	222 (85.1)	0.51
Mean age(SD)	38 (13.4)	37.7 (14.1)	0.83
Illiterate (%)	204 (59.3)	163 (62.4)	0.43
Socioeconomic status of HH (%)			
Poorest	109 (31.7)	101 (38.7)	
Middle	98 (28.5)	80 (30.7)	**0.05**
Richest	137 (39.8)	80 (30.7)	
Migrated to Delhi	197 (57.3)	141 (54)	0.42
Staying the same locality for >5 yrs (%)	263 (76.5)	196 (75.1)	0.169
Distance to nearest public healthcare facility (<5 kms) (%)	275 (80)	207 (79.3)	0.497
Facility visited for MCH services (%)			
Public	262 (76.2)	201 (77)	0.304
Private	59 (17.2)	36 (13.8)	
NGO	5 (1.5)	3 (1.2)	
Others	18 (5.2)	21 (8)	
Ration card	152 (44.2)	113 (43.3)	0.758
BPL	99 (28.8)	70 (26.8)	0.27
RSBY	83 (24.1)	45 (17.2)	**0.05**

**Table 2:** **Profile and pregnancy details of the study population**

**Characteristics**	**CPW (n = 184)**	**RDW (n = 160)**
Age in years (SD)	23.4 (3.9)	24.7 (4.36)
Duration of pregnancy for CPW	6.3 (1.7)	NA
Illiterate (%)	88 (47.8)	88 (55)
Homemaker	180 (97.8)	156 (97.5)
Marriage after 18 yrs	161 (87.5)	136 (85)
Pregnancy after 18 yrs*	173 (95)	149 (95)
First pregnancy	64 (34.8)	41 (25.6)

*n for CPW = 182 n for RDW =157.

### Availability of ANC and other health care services

All three clusters had access to some kind of health care facility (government/private) for maternal, neonatal and child health (MNCH) care within 2.5 kilometres (km) radius. One referral hospital was situated at a distance of 5kms and several other referral hospitals beyond 10 km. In two out of three clusters, no private nursing home was present. A Government-run MCH centre and Rashtriya Swasthya Bima Yojana (RSBY) (a public health insurance scheme for people belonging to unorganized sector) empanelled hospital was present within each of two clusters. The Primary Urban Health Centre (PUHC) was situated within one of the slum clusters. A total of 17 private clinics (registered and unregistered) and one laboratory was present in the study area. Though 28% of the women reported presence of any community health worker in their area, only one pregnant woman reported home visits by a health worker.

### Utilization of ANC

Of the 344 women 70(20.6%, 95% CI 16, 25) did not avail any kind of ANC services. A detailed description of ANC service utilization is provided in Table 3. Out of 274 women who availed ANC services, 44% (95% CI 38, 50) registered for ANC in the first trimester. The univariable analysis comparing women who availed ANC with those who did not, showed association with HH factors viz. socioeconomic status, religion, belonging to Delhi, and duration of stay in the locality and individual factors namely schooling, age at marriage and parity (Table 4). On multivariable analysis adjusted for rest of the factors, richer households (OR 2.13 [95% CI 0.98, 4.62]), being resident of the locality for a duration >5 years (OR 2 [95% CI 1.06, 3.77]), women’s schooling (OR 2.15 [95%CI1.1, 4.17]) and first pregnancy (OR 2.58 [95%CI 1.19, 5.58]) were strongly associated to availing ANC services at a facility.

**Table 3:** **Information on ANC received**

**Services received**	**N = 344**
Some kind of ANC	273 (79.36)
Did not avail ANC at a facility	71 (20.6)
Availed ANC at facility (n = 273)	
Public facility	197 (72.2)
Private facility	59 (21.6)
NGO	17 (6.2)
% who registered for ANC (n = 272) in	
First trimester	119 (43.8)
Second trimester	135 (49.6)
Third trimester	18 (6.6)
Received prescription for	
Iron tablet	242 (70.4)
From public hospital	178 (73.9)
Reported to consume regularly	191 (78.9)
Calcium	245 (71.43)
From public hospital	196 (80)
Reported to consume regularly	172 (72.6)
TT received (n = 170)	143 (84.1)
Aware about ICTC (n = 342)	128 (37.4)
Visited ICTC (n = 128)	
Both husband and wife	21 (16.4)
One of them	79 (61)
Did not visit	28 (22.6)

**Table 4:** **Association of socio demographic profile and availing ANC**

**Characteristics**	**Availed ANC (n = 274)**	**Did not avail ANC (n = 70)**	**Crude OR (95% CI), p value**	Adjusted OR* (95% CI), p value
Family size	5.5 (2.70)	5.3 (2.65)	1.03 (0.93, 1.14)	1 (0.87, 1.14)
			0.47	0.98
Socio-economic Scale (SES) of HH**				
Poorest	71 (26.01)	38 (53.52)	1	1
Middle	82 (30.04)	16 (22.54)	2.74 (1.41,,5.33)	2.29 (0.98, 4.62)
Richest	120 (44.1)	17 (22.94)	3.77 (1.98, 7.18)	2.13 (0.40, 2.1)
			0.001	0.039
Nuclear family	173 (63.4)	52 (73.24)	0.63 (0.35, 1.13)	1.21 (0.58,2.55)
			0.12	0.6
Non-Muslim	73 (26.74)	17 (23.94)	1.16 (0.63, 2.12)0.63	1.1 (0.54, 2.26)
				0.77
Schooling of Head of Household	107 (39.2)	20 (28.2)	1.64 (0.92, 2.91)	1.23 (0.62, 2.4)
			0.08	0.54
Schooling of the woman	150 (55)	18 (25.35)	3.59 (2, 6.45)	2.15 (1.10,4.18)
			<0.001	0.016
Belong to Delhi	124 (45.42)	23 (32.4)	1.73 (1.001, 3.01)	1.23 (0.67, 2.3)
			0.046	0.49
Longer duration of stay in the locality (>5 years)	221 (81)	42 (59.2)	2.94 (1.67, 5.14)	2 (1.06, 3.77)
			<0.001	0.032
Age at marriage (%) >18 years	241 (88.28)	56 (78.9)	2.02 (1.02 3.97)	1.65 (0.78, 3.52)
			0.043	0.18
First pregnancy /child	95 (34.8)	10 (14.1)	3.26 (1.59, 6.64)	2.58 (1.19, 5.58)
			0.0004	0.016

*Adjusted using multiple logistic regression method; **The SES scale is a composite of house type, floor, house ownership, separate kitchen, TV, Refrigerator, mobile phone, washing machine, total HH income, number of rooms by principal component analysis.

### Services and counselling received during ANC

The medical care as a part of ANC were reported to be done in more than 80% of the women availing ANC (Table 5). However, when a correlation was seen between the components, it was moderate, except for correlation between blood and urine examination and iron and folic acid (IFA) and calcium prescription which was high (not shown in the table). Regular intake of IFA and calcium tablets respectively, was reported by 78.9% and 72%. The common reasons cited for erratic intake were “Just don’t feel like” (33% for IFA and 41% for calcium), “Felt uneasy” (33% for IFA and calcium), “Gastric trouble” (19% for IFA and 12% for calcium). The counselling received during ANC visits for various issues listed in the table varied between 35-80% with counselling on themes like early initiation of breast feeding, exclusive breast feeding (EBF) and family planning (FP) reported less frequently. Additional counselling on managing frequently occurring symptoms was reported by only 30%. In general a higher proportion of RDW reported receiving counselling services as compared to PW.

**Table 5:** **Services and counselling received during ANC**

	**Overall (n = 279)**	**CPW (n = 144)**	**RDW (n = 135)**
**Medical care**			
Past history was asked	227 (81.4)	117 (81.3)	110 (81.5)
Physical examination	245 (87.8)	124 (86.1)	121 (89.6)
Body weight measured	235 (84.2)	120 (83.3)	115 (85.2)
Blood Pressure measured	247 (88.5)	126 (87.5)	121 (89.6)
Blood test done	229 (82)	110 (76.4)	119 (88.2)
Urine test done	226 (81)	107 (74.3)	119 (88.2)
IFA prescription given	230 (82.4)	113 (78.5)	117 (86.7)
Calcium prescription given	232 (83.2)	113 (78.5)	119 (88.2)
**Counselling on ANC**			
Regular follow-up	198 (71)	94 (65.3)	104 (77.1)
Hospital birthing	170 (60.9)	82 (56.9)	88 (65.2)
Importance of TT	217 (77.8)	104 (72.2)	113 (83.7)
Regular IFA	218 (78.1)	107 (74.3)	111 (82.2)
Diet	164 (58.8)	70 (48.6)	94 (69.6)
Breast feeding initiation	110 (39.4)	41 (28.5)	69 (51.1)
Exclusive breast feeding	118 (42.3)	45 (31.3)	73 (54.1)
Family planning	104 (37.3)	43 (29.9)	61 (45.2)
**Counselling on dealing with symptoms**			
Vomiting	86 (30.82)	38 (26.4)	48 (35.6)
Constipation	49 (17.6)	23 (15.9)	26 (19.3)
Gastric fullness	53 (19)	26 (18.1)	27 (20)
Pain	55 (19.8)	26 (18.2)	29 (21.5)
Swelling	56 (20.1)	26 (18.2)	30 (22.2)
Headache	67 (24.1)	29 (20.1)	38 (28.2)
Bleeding	72 (25.8)	33 (22.9)	39 (28.9)
Fatigue	83 (29.8)	37 (25.7)	46 (34.1)

(CPW: Currently Pregnant Women, RDW: Recently Delivered Women, ANC- Antenatal care, TT: Tetanus Toxoid, IFA: Iron and Folic Acid).

### Satisfaction with the ANC services

**Figure 2:**
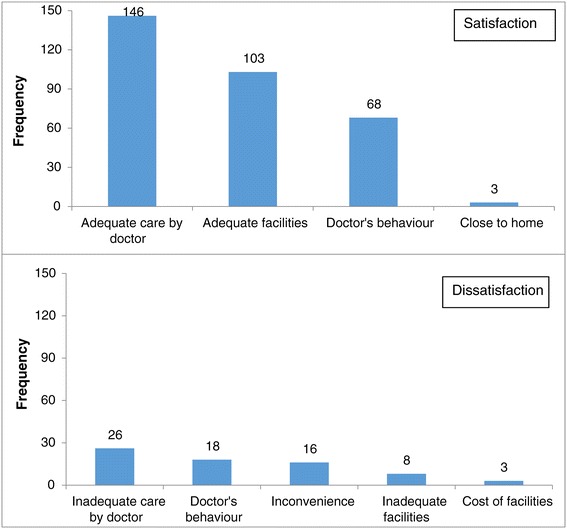
**Reasons for satisfaction and dissatisfaction with the ANC services received perception of the women.**

About 68% respondents reported that they were satisfied with the ANC services availed. As seen in Figure 2 the key reason for satisfaction and dissatisfaction was based on their perception of quality of care and behaviour of the care provider.

### Dietary and nutrient intake

The mean nutrient intake compared with the RDA for sedentary activity in PW and RDW is shown in Table 6. The majority of PW and RDW had an inadequate nutrient adequacy ratio NAR (<0.66) with respect to protein, and micronutrients like iron, calcium, vitamin A, vitamin C, thiamine, riboflavin, niacin, zinc and vitamin B12. One third of women also had an inadequate intake of energy (RDW (34.2%), PW (36%)).

**Table 6:** **Nutrient intake and Nutrient Adequacy Ratio (NAR) of the study population**

**Nutrient**	**Pregnant women(n = 178)**			**Nutrient Adequacy Ratio**			**Recently delivered women (n = 155)**			**Nutrient Adequacy Ratio**		
	**RDA (2010)**	**Mean intake ± SD**	**% adequacy**	**Inadequate (<.66 of RDA) n(%)**	**Fairly adequate (.66- < 1.00 of the RDA) n(%)**	**Adequate (≥1) n(%)**	**RDA(2010)**	**Mean intake ± SD**	**% adequacy**	**Inadequate (<.66 of RDA) n(%)**	**Fairly adequate (.66- < 1.00 of the RDA) n(%)**	**Adequate (≥1) n(%)**
Energy (kcal)	2250*	2021 ± 734	89.8	-	-	-	2500*	2277 ± 757	91.1	-	-	-
Protein (g)	82.2	53.3 ± 21.9	64.8	**106 (59.6)**	57 (32.0)	15 (8.4)	77.9	63.4 ± 20.9	81.4	44 (28.4)	79 (51.0)	32 (20.6)
Fat (g)	atleast 20 E%**	71.6 ± 40.1	143.2	-	-	-	atleast 20 E%**	79.1 ± 45.8	158.2	-	-	-
Iron (mg)	35	12.0 ± 5.8	**34.3**	**170 (95.5)**	8 (4.5)	0	25	14.0 ± 5.8	56	**111 (71.6)**	40 (25.8)	4 (2.6)
Calcium (mg)	1200	568.0 ± 370.2	**47.3**	**145 (81.5)**	26 (14.6)	7 (3.9)	1200	634 ± 441	52.8	**109 (70.3)**	33 (21.3)	13 (8.4)
Folic acid (total) (mcg)	500	115 ± 60.5	**23**	**177 (99.4)**	1 (.6)	0	300	144.0 ± 78.3	**48**	**132 (85.2)**	16 (10.3)	7 (1.6)
Vitamin A (mcg)	800	266 ± 211.2	**33.3**	**160 (89.9)**	15 (8.5)	3 (1.7)	950	330 ± 309	**34.7**	**131 (84.5)**	17 (11.0)	7 (4.5)
Vitamin C (mg)	60	44 ± 38	73.3	**107 (60.1)**	30 (16.9)	41 (23.0)	80	31.0 ± 30.0	**38.8**	**135 (87.1)**	12 (7.7)	8 (5.2)
Thiamin (mg)	1.2	1.1 ± 0.6	91.7	57 (32.0)	56 (31.5)	65 (36.5)	1.3	1.4 ± 0.6	107.7	25 (16.1)	58(37.4)	72 (46.5)
Riboflavin (mg)	1.4	0.8 ± 0.4	57.1	**117 (65.7)**	45 (25.3)	16 (9.0)	1.5	0.96 ± 0.5	64	**85 (54.8)**	46 (29.7)	24 (15.5)
Niacin (mg)	14	10 ± 5.4	71.4	**94 (52.8)**	54 (30.3)	30 (16.9)	16	11.0 ± 5.5	68.8	77 (49.7)	54 (34.8)	24 (15.5)
Zinc (mg)	12	5.5 ± 2.4	**45.8**	**150 (84.3)**	26 (14.6)	2 (1.1)	12	6.3 ± 2.50	**52.5**	**123 (79.4)**	28 (18.1)	4 (2.6)
Vitamin B12 (mg)	1.20	0.54 ± 0.68	**45**	**140 (78.7)**	19 (10.7)	19 (10.7)	1.5	1.50 ± 6.31	100	**97 (62.6)**	21 (13.5)	37 (23.9)

*Estimated Average Requirement (EAR) for Energy **% contribution of fat calories in the total energy intake **Bold**: Indicates that >50% of women had inadequate intake.

A comparison of dietary adequacy in women who availed ANC versus those who did not, showed no association, except for protein intake which was lower in women who did not avail ANC (p = 0.019). However, these results need to be interpreted with caution owing to the multiple hypotheses tests done in a relatively small sample. The food consumption pattern based on the food frequency questionnaire (FFQ) showed a daily consumption of cereals, vegetables, milk, fats and sugar. However, when compared with the quantitative estimates from the 24 hour recall data, the mean consumption of vegetables (10%) and milk (60%) were below recommended intakes. Majority of the women reported consuming fruits, green leafy vegetables, pulses/legumes and flesh foods only 1–2 times a week with a low mean consumption per day as compared to recommended intakes. The commonly consumed packaged food products both labelled and unlabelled, included *rusk* (baked crackers) (89%), biscuits (76%) and *bhujia,* an Indian savoury fried snack made with chickpea flour (60%). These products are known sources of saturated fat, trans-fats, energy and salt [25,26].

### Nutritional status assessment

After removing the outliers, 59% of PW in the second trimester whose repeat weights were available after a 2 week interval (n = 66) had a weight gain of less than 60 g /day (as per recommendations) and 53% of PW in third trimester (n = 60) had a weight gain of less than 54 g/day (as per recommendations). There was no association between weight gain patterns during the second and the third trimester of pregnancy and availing ANC (p = 0.27; p = 0.45). Among the RDW, 16% had CED as estimated by a BMI of less than 18.5. The prevalence of any anaemia was 85% among PW and 97.1% in RDW. The prevalence of severe anaemia was 18% (95% CI 12, 24) and 15% (95% CI 9, 22) in PW and RDW, respectively. There was no statistically significant difference in mean haemoglobin levels between those who availed ANC and those who did not.

## Discussion

This survey from the urban slums of Delhi, highlights various facets of ANC services offered, dietary intake patterns and nutritional status of pregnant women and lactating mothers. Though most of the women surveyed received some kind of antenatal care, the complete ANC package especially the counselling component was observed to be inadequate. The nutrient intake in the study population, showed suboptimal consumption of micronutrients, inadequate weight gain and high prevalence of nutritional anaemia irrespective of ANC received. The women who availed ANC were mostly satisfied with the care they received.

In our study population 20% of women did not avail any ANC services at a health facility which is almost similar to the NFHS-3 Delhi slum data (25%), but higher when compared to a slum survey from Mumbai where only 7% of women did not avail any ANC [27]. In our study the determinants for non-utilization were poverty, literacy, migration, duration of stay in the locality and high parity which was similar to a study using NFHS data [28] and the determinants for home delivery in this population [17].

The women we interviewed reported clinical examination, IFA and calcium prescription and TT vaccination as services provided under ANC. Partial ANC service provision was reported in a study in Agra district of Uttar Pradesh where more than 50% were prescribed IFA and received TT but only a third of the pregnant women underwent abdominal examination and had BP measurements taken [29]. Only a fourth of our study population reported receiving counselling on diet and exclusive breast feeding. Our data also suggest that counselling was provided more towards the later stages of pregnancy as this service provision was reported more often by RDWs than PWs. This could limit the gains expected out of these sessions in bringing about behaviour change that is most critical in the earlier stages of pregnancy to achieve a greater impact on health outcomes.

The dietary intake especially protein and micronutrient intake was sub-optimal. The mean energy intake of our study population was higher than reported in other studies in urban poor settlements of Delhi [15,16]. However, micronutrient intakes especially of iron and vitamin A were lower in a higher proportion of our study population as compared to the study by Kapil et al. [15]. The quantitative estimates of consumption of micronutrient rich food sources like green leafy vegetables and seasonal fruits were low, while the consumption of unhealthy sources of fats and energy were high. Low cost dietary sources of micronutrients were available in the food environment of the community under study. However, culturally dictated food fads and nutritional taboos during pregnancy [30] might have prevented the women from consuming locally available low cost food. Further, despite the prescription and availability of nutritional supplements like IFA and calcium, a high prevalence of iron deficiency was recorded. All these findings point to the compelling and vital role that nutritional counselling can play during ANC visits to promote locally available, affordable micronutrient rich food sources. This along with reinforcement for compliance to nutritional supplements, may effectively address adverse health outcomes like anaemia and inadequate weight gain during pregnancy. As a corollary, this opportunity, if missed, could potentially retard efforts to improve maternal and child health despite the intensive resource inputs that are noticeably being allocated for this purpose.

In the present study, 75% women availed ANC from a public health facility in contrast to the Mumbai survey where an equal proportion of women availed the public and private sector health facilities [27]. The NFHS 3 data on ANC utilization cites the reasons for preferential utilization of private health care over public facilities (for those who can afford it) as being due to ineffective outreach, overcrowding, and poor quality of services in the urban public health system [28]. Studies from sub-Saharan Africa have also documented the severe shortage of health workers in hindering the capacity of health systems to deliver the required services [31]. Keeping in view the dependence of the urban poor on the overburdened and overcrowded public health facility, the counselling component may perhaps be effectively provided through the continuum of care approach [1] and utilizing the trained community based health workers. Studies from sub-Saharan Africa and Bangladesh have shown that health extension programs aiming to increase access to and equity in essential health care through community based outreach health services at the doorsteps of the residents have contributed in improving contraceptive use and utilization of some components of maternal health services [32-35]. We observed a very nominal presence of health workers in the community. Under the Delhi State Health Mission, the community health workers called ASHAs (Accredited Social Health Activists) are being recruited and trained to provide community based maternal and child health services in urban slums. Though health promotion is one of the important mandates for this cadre, capacity building for the specialized skills needed to provide effective counselling is much desirable. A systematic review has underscored the effectiveness of nutrition training for health workers in improving care providers’ child feeding practices in terms of feeding frequency, energy intake, and dietary diversity of children aged six months to 2 years [36]. Through the ANCHUL project, we intend to explore the effectiveness of utilizing community health workers i.e. ASHAs in decentralizing the workload of health promotion which would lead to effective sharing of counselling services between health facilities and community health workers. Further research on this aspect in the urban Indian context is required to demonstrate the effectiveness of role of ASHAs in nutrition counselling and improving health outcomes.

Some of the potential limitations in this study were (1) all information on ANC services received were based on recall which might have led to recall bias; (2) dietary intake data were collected for only 2 days in one particular season, which might not be representative of the habitual dietary intake of the population; (3) weight gain pattern measured needs to be interpreted with caution as weights were measured only at two time points, a fortnight apart; and (4) the sampling frame was only from 3 slum clusters from one of the districts of Delhi, which might not be representative of entire urban poor population.

### Key messages

- Women in the urban poor settlements of Delhi accessed some form of ANC but the complete package of care especially the counselling component was grossly inadequate.
- Irrespective of the ANC received, the dietary intake of pregnant women was suboptimal, with poor weight gain and high prevalence of anemia.
- There is a need for strengthening the nutrition counselling component of ANC.
- Empowering community based health care workers in providing effective nutrition counselling should be explored given our overburdened public health system.

## Abbreviations

ANC: Antenatal care
WHO: World health organization
IEC: Information education and communication
PW: Pregnant women
RDW: Recently delivered women
HH: Households
RDA: Recommended dietary allowance
NAR: Nutrient adequacy ratio
BMI: Body mass index
CED: Chronic energy deficency
PUHC: Primary urban health centre
RSBY: Rashtriya Swasthya Bima Yojana
FP: Family planning
FFQ: Food frequency questionnaire
EBF: Exclusive breast feeding ASHA, Accredited social health activist
ANCHUL: Antenatal and child health care in urban slums
TT: Tetanus toxoid
IFA: Iron and folic acid
